# Effect of Grinding and Polishing Protocols on Surface Roughness, Flexural Strength, and Phase Transformation of High-Translucent 5 mol% Yttria-Partially Stabilized Zirconia

**DOI:** 10.1055/s-0044-1787001

**Published:** 2024-06-28

**Authors:** Chatnarong Phatphutthitham, Boondarick Niyatiwatchanchai, Phakvalunch Rujiraprasert, Junji Tagami, Thanaphum Osathanon, Anucharte Srijunbarl, Thawanrat Singthong, Sarat Suriyasangpetch, Dusit Nantanapiboon

**Affiliations:** 1 Department of Operative Dentistry, Faculty of Dentistry, Chulalongkorn University, Bangkok, Thailand; 2 Faculty of Dentistry, Chulalongkorn University, Pathumwan, Bangkok, Thailand; 3 Center of Excellence for Dental Stem Cell Biology, Department of Anatomy, Faculty of Dentistry, Chulalongkorn University, Bangkok, Thailand; 4 Dental Material Research and Development Center, Faculty of Dentistry, Chulalongkorn University, Bangkok, Thailand; 5 Department of Advanced General Dentistry, Faculty of Dentistry, Mahidol University, Bangkok, Thailand.; 6 Clinic of General, Special Care, and Geriatric Dentistry, Center for Dental Medicine, University of Zurich, Zurich, Switzerland

**Keywords:** zirconia, 5Y-PSZ, surface roughness, biaxial flexural strength

## Abstract

**Objectives** This study evaluated surface roughness, biaxial flexural strength, and phase transformation of 5Y-PSZ after grinding and polishing with different protocols.

**Material and Methods** Two commercial 5Y-PSZ, Lava Esthetic (L) and Cercon xt (C), were used and divided into 3 groups: LC and CC represented unpolished control groups; LE and CE were polished with protocol I (EVE DIASYNT® PLUS HP following with EVE DIACERA RA); and LJ and CJ were polished with protocol II (Superfine diamond bur following with Jota ZIR Gloss polishing kit). Surface roughness was evaluated after polishing step-by-step with a contact-type profilometer. After high-gross polishing, the specimens were subjected to biaxial flexural strength test, crystallographic microstructure analysis using an X-ray diffractometer (XRD), and surface micro-topography using scanning electron microscopy (SEM).

**Statistical Analysis** Surface roughness differences after each step and biaxial flexural strength between groups were evaluated with one-way ANOVA, followed by Bonferroni post-hoc analysis. Changes in surface roughness across four different time points within groups were assessed using one-way repeated measures ANOVA, followed by Bonferroni post-hoc analysis.

**Results** After high-gross polishing, both polishing protocols showed significantly lower surface roughness than the grinding group ( *p* < 0.05). The LE and CE groups exhibited the highest surface roughness values, which were significant differences from the LJ and CJ groups ( *p* < 0.05). The LE group showed significantly lower biaxial flexural strength compared to the LC group ( *p* < 0.05). However, there was no statistically significant difference in the CE and CJ groups compared to the control group ( *p* > 0.05). Furthermore, all polishing protocols did not change the phase transformation of zirconia.

**Conclusion** Polishing protocol II provided a smoother surface than the protocol I after high-gross polishing, while the biaxial flexural strength of materials remained unaffected.

## Introduction

Zirconia is an alternative material in restorative dentistry due to its superior mechanical properties, biocompatibility, and esthetic. 1 2 Zirconia is a polymorphic metastable material consisting of three phases, namely, monoclinic phase ( *m* -ZrO _2_ ), tetragonal phase ( *t* -ZrO _2_ ), and cubic phase ( *c* -ZrO _2_ ). Due to the unstable tetragonal phase, 3 mol% yttrium was typically added to form 3 mol% yttria-tetragonal zirconia polycrystal (3Y-TZP). However, the 3Y-TZP mainly contains the tetragonal phase, which can be transformed into a monoclinic phase under stress application conditions, called phase transformation toughening. The phase transformation phenomenon in 3Y-TZP is associated with a volumetric expansion (3–5%) of the tetragonal phase to the monoclinic phase, which induces compressive stresses that eliminate crack propagation. 3

Despite the exceptional mechanical properties of 3Y-TZP, the major drawback is its low translucency. Consequently, highly translucent monolithic zirconia has been modified by increasing the yttria content to 5 mol% of yttrium oxide to form the cubic phase up to 53% and decreasing the content of the alumina (Al _2_ O _3_ ) dopant, known as 5 mol% yttria-partially stabilized zirconia or 5Y-PSZ. 4 The component of 5Y-PSZ is formed with a mixed cubic/tetragonal content. 5 Cubic grains are larger than tetragonal grains, contributing to a lower grain boundary between the zirconia crystals and leading to higher translucency. Since the fraction of the cubic phase increases, the amount of the tetragonal phase is reduced. Therefore, the mechanical strength of 5Y-PSZ is also decreased to almost half of that of 3Y-TZP. 6

5Y-PSZ is commonly used for dental crowns and partial-fixed prostheses. After the restoration cementation, adjustment is another crucial step to create a proper contour and occlusion. The smooth surface of the restoration prevents antagonist wear and plaque deposition that could compromise the longevity of the restoration. 7 In clinical procedures, glazing and polishing are commonly performed to achieve a smooth restoration surface. Several studies reported that the surface roughness of zirconia restoration from glazing and polishing was comparable. 8 9 10 However, the glazed surface is commonly worn off after use over time; it can exhibit significantly higher surface roughness than the polished surface. 11 12 Therefore, the polishing procedure is more practical and applicable in clinical situations.

Due to the high mechanical strengths and hardness of zirconia, zirconia polishing systems have been widely introduced to the market, which improves the diamond particle coating in the grinding burs. 13 Coarse-grit and fine-grit diamond burs are used for polishing zirconia. 14 Polishing the 3Y-TZP following the company guideline, from a coarse finishing diamond bur to a fine silicone-impregnated diamond bur, had the lowest surface roughness. 15 A stone-grinding bur could also be used to grind and polish zirconia. Previous studies reported that grinding 3Y-TZP with a stone grinding bur without water coolant exhibited lower surface roughness and phase transformation. 14 16 Furthermore, various polishing systems such as diamond rotary instruments, stone grinding burs, silicone rubber disks, and silicon carbide or aluminum oxide–coated abrasive disks showed similar surface roughness and did not exhibit phase transformation. 9 17

Furthermore, the parameters during clinical adjustment, such as heat, pressure, and force, could alter the surface structure, generate a stress-induced transformation of zirconia, and affect the mechanical properties. 13 18 There are limited studies on the influence of grinding and polishing with different grinding burs on the 5Y-PSZ. Therefore, this study aimed to evaluate the effect of two grinding and polishing protocols on surface roughness and biaxial flexural strength of 5Y-PSZ. The null hypothesis was that finishing polishing with two grinding and polishing protocols would not change the surface roughness and flexural strength of 5Y-PSZ.

## Materials and Methods

### Specimen Preparations

All materials used in this study are presented in Table 1 . Two commercial 5Y-PSZ products, Lava Esthetic (3M ESPE, St Paul, MN, United States) and Cercon xt (DeguDent GmbH, Hanau-Wolfgang, Germany), were used to fabricate the specimens.

**Table 1:** Materials, grinding, and polishing burs used in the study.

Products	Particle size	Batch number	Manufacturer
Lava Esthetic	5mol% yttrium oxide	REF 69321 LOT 7312198	3M ESPE, Minnesota, United States
Cercon xt	Yttrium oxide 9% Hafnium oxide <3% aluminum oxide and silicon oxide <1%	REF 53 6611 1114 Batch code LOT 18037374	DeguDent GmbH, Hanau-Wolfgang, Germany
**Protocol I**			
EVE DIASYNT PLUS HP	Organic synthetic stone diamond particle sized 150–200 μm	REF 7784	EVE Ernst Vetter GmbH, Pforzheim, Germany
EVE DIACERA RA Twist medium	Diamond particle sized 15–20 μm	REF 7684 LOT 433138	EVE Ernst Vetter GmbH, Pforzheim, Germany
EVE DIACERA RA Twist fine	Diamond particle sized 4–8 μm	REF 7784 LOT430937	EVE Ernst Vetter GmbH, Pforzheim, Germany
**Protocol II**			
Jota Z850.FG	Diamond particle sized 38–45 μm	LOT 569103	Jota, Rüthi, Switzerland
Jota ZIR Gloss Chairside Set medium	Diamond particle sized 15–20 μm	LOT 807227	Jota, Rüthi, Switzerland
Jota ZIR Gloss Chairside Set fine	Diamond particle sized 4–8 μm	LOT 618343	Jota, Rüthi, Switzerland

The specimen was designed using a computer-aided design/computer-aided manufacturing (CAD/CAM) system with Autodesk Fusion 360 program (Autodesk Inc., San Francisco, CA, United States). Partially sintered yttrium-stabilized zirconia blanks from each brand were milled into disk-shaped specimens measuring 18 mm in diameter and 1.2 mm in height for the control groups. For the testing groups, disk-shaped specimens of the same dimensions as the control group were prepared, with an additional design featuring a simulated high-contact area measuring 3.6 mm in diameter and 0.6 mm in height at the center of each disk. All specimens were fabricated by a five-axis milling machine (inLab MC X5, Dentsply Sirona, Bensheim, Germany). The specimens were sintered in a sintering furnace (inFire HTC speed, Dentsply Sirona, Bensheim, Germany) with a heating and cooling time rate of 20°C per minute until reaching 800°C. Then, the temperature was continuously increased to 1,500°C to be sintered-holding time of 2 hours according to the manufacturer's instruction. Volumetric shrinkage compensation was at 20% after sintering.

**Fig. 1:**
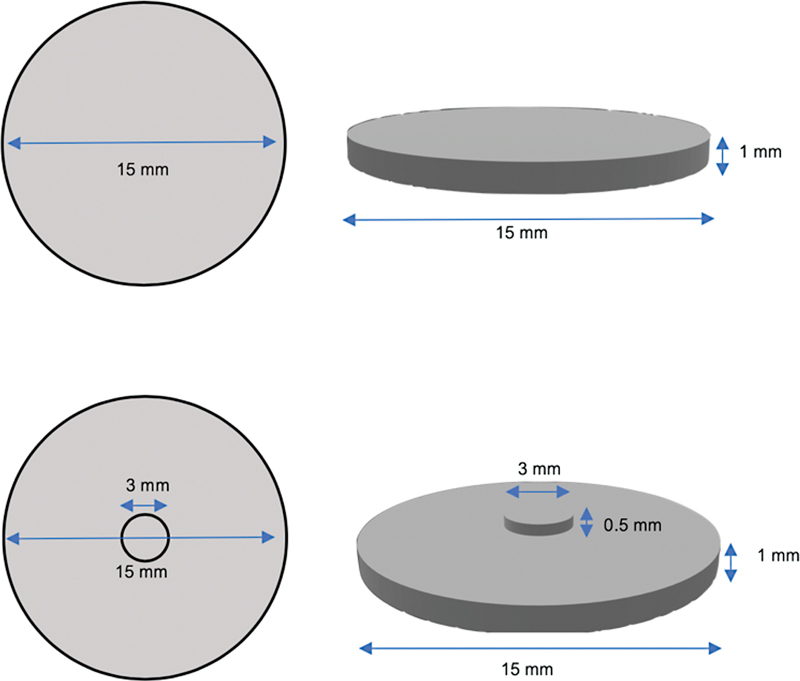
The dimensions of the testing and control groups.

The sintered specimens were polished with 320-, 600-, 1,200-, and 2,000-grit silicon carbide papers (TOA CO. LTD., Osaka, Japan) on a polishing machine (NANO2000, PACE Technologies, Tucson, AZ, United States) at a speed of 300 rpm for 15 seconds with continuous water irrigation. The specimen dimensions were verified with a digital vernier caliper (Mitutoyo, Kanagawa, Japan). The final dimensions of all specimens were 15 ± 0.2 mm in diameter and 1 ± 0.2 mm in height, following the ISO 6872:2015 specimen preparation guideline for biaxial flexural strength tests for ceramic materials. 19 Moreover, the simulated high-contact area of the tested groups was verified to be 3 ± 0.2 mm in diameter and 0.5 ± 0.2 mm in height at the center of each disk. The dimensions of the testing and control groups are presented in Fig. 1 .

### Grinding and Polishing Protocols

Sixty-six specimens were divided into six groups, i.e., LC and CC (unpolished), LE and CE (protocol I), and LJ and CJ (protocol II), following the grinding and polishing protocols in Table 2 . A custom-made polishing machine was used to control the speed, direction, and application force during the procedures. The grinding and polishing burs were mounted on the machine, ground, and polished the specimen in a continuously sweeping motion within a 5-mm distance at a rate of 1 mm/s with an applied force of 0.98 N 20 21 and 2 N 22 23 on the grinding and polishing burs, respectively.

**Table 2:** Grinding and polishing protocols.

Protocol	Group	Grinding and polishing bur	Speed (rpm)	Cycle	Water coolant	Applied force (N)
I	EVE Diasynt Plus (LE and CE)	EVE DIASYNT PLUS HP	10,000	40	No	0.98
		EVE DIACERA RA W14DCmf	10,000	40	No	2
		EVE DIACERA RA W14DC	10,000	40	No	2
II	Jota (LJ and CJ)	Jota Z850.FG.018	160,000	40	Yes	0.98
		Jota ZIR9861M.RA.040	10,000	40	No	2
		Jota ZIR9863F.RA.140	10,000	40	No	2

**Fig. 2:**
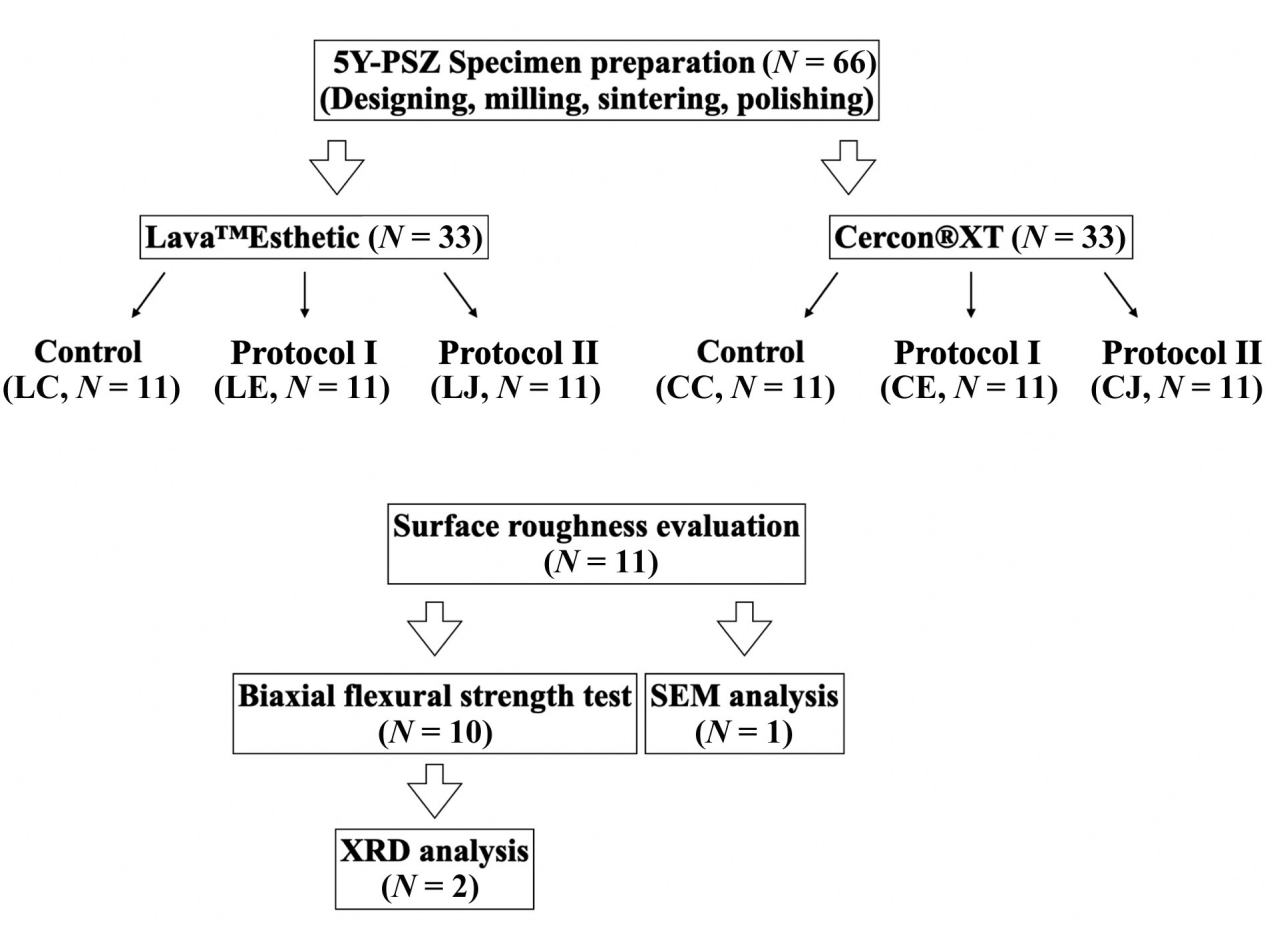
Flowchart of the experimental procedures.

The sequence of grinding and polishing procedures with a diamond stone bur and a superfine diamond bur is shown in Fig. 2 . The group with diamond stone bur (Protocol I) was performed with a low-speed handpiece (NSK Nakanishi Inc., Tochigi, Japan). In the grinding procedure of fine diamond bur (Protocol II), a high-speed handpiece (NSK Nakanishi Inc.) was used with copious water coolant irrigation. The polishing was subsequently performed using medium and fine diamond-impregnated silicone burs with a low-speed handpiece. The burs were renewed every four specimens. The specimens were rinsed with distilled water, ultrasonically cleaned for 3 minutes, and air dried before surface roughness evaluation. Surface roughness values were measured after each step as baseline (Ra0), after grinding (Ra1), after polishing (Ra2), and after high-gloss polishing (Ra3).

After surface roughness evaluation, the specimen of all groups was randomly selected and stored in a desiccator for 24 hours before undergoing evaluation of microscopic surface topography by using scanning electron microscopy (SEM) and phase analysis by using X-ray diffractometry (XRD).

### Surface Roughness Evaluation

The surface roughness values of all specimens were evaluated from a contact-type profilometer (Talyscan 150, Taylor Hobson Ltd., Leicester, England) with a 5-μm diamond stylus tip. The specimens were fixed on the fixation stand and the stylus was adjusted to be in the position with a contact area of 2 × 2 mm ^2^ on a specimen surface. A constant load of 5 N with a speed of 1,500 μm/s was applied. The measurements were performed with five parallel lines before calculating the mean surface roughness value.

### Biaxial Flexural Strength Test

The biaxial flexural strength test was performed using a universal testing machine (Servo Hydraulic system Model 8872, Instron, Buckinghamshire, England). Each specimen was placed with the polished side facing down toward the support balls (tension side), with three steel balls of 3.2-mm diameter positioned 120 degrees apart from each other, forming a 12.0-mm diameter circular platform. A loading force was applied with a 1.0-mm tip positioned at the center of the specimen with a crosshead speed of 0.5 mm/min. The maximum fracture load of each specimen was recorded in newtons and calculated to the biaxial flexural strength in megapascals. 19

### Scanning Electron Microscopy Analysis

The microscopic surface topography was evaluated using a scanning electron microscope (TM3000 Tabletop microscope, Hitachi-High Technologies, Tokyo, Japan). A randomly selected specimen in each group ( *n* = 1) was mounted on a metallic cylinder and sputtered with a thin coat of Au-Pt using a fine coater (JFC-1200, Jeol, Tokyo, Japan). The SEM was operated at 20 kV with 3,000x and 10,000x magnifications.

### X-Ray Diffraction Analysis

A quantitative analysis of phase transformation was performed using X-ray diffraction (XRD) analysis (Bruker D8, Cambridge, MA, United States) and the DIFFRAC.EVA analysis program version 2 (Bruker AXS GmbH, Karlsruhe, Germany). Specimens were randomly selected in each group ( *n* = 2) to determine the composition of zirconium oxide crystals and the phase transformation by a peak intensity ratio from the XRD patterns. Structural studies of crystal phase transformation were performed using XRD. Diffraction patterns were obtained using Cu-Kα radiation (λ = 1.5406 Å) in the range of 20 to 40 degrees of 2θ with a step size of 0.01 and a step duration of 50.165 seconds. The peaks were refined using the pattern-decomposition and profile fitting functions of the HighScore Plus software (Malvern Panalytical, Worcestershire, United Kingdom). The phase structure determination and refinement were performed using Rietveld refinement with TOPAS V3.0 software (Bruker, Karlsruhe, Germany).

### Statistical Analysis

All statistical tests were analyzed by using an IBM SPSS software version 28.0 for Mac (IBM Corp., Armonk, New York, United States). The results of statistical analyses with *p* -value less than 0.05 were interpreted as statistically significant. Normal distribution was determined using the Shapiro–Wilk test. Homogeneity of variances was done using Levene's test. Difference of surface roughness after each step and biaxial flexural strength between groups was determined using one-way analysis of variance (ANOVA), followed by Bonferroni post hoc analysis. Difference of surface roughness among four different time points within group was evaluated using one-way repeated measure ANOVA followed by Bonferroni post hoc analysis.

## Results

### Surface Roughness Evaluation

The statistical analysis revealed that all Ra groups followed a normal distribution, as determined by the Shapiro–Wilk test. The mean of Ra and *p* -values of both 5Y-PSZ at baseline (Ra0), after grinding (Ra1), after polishing (Ra2), and after high-gloss polishing (Ra3) are shown in Table 3 . All groups revealed a similar pattern of surface roughness values and in the Ra0 there was no significant difference. The roughness values were significantly increased in Ra1 ( *p* < 0.05) and mainly decreased in Ra2 and Ra3 ( *p* < 0.05) in all groups. However, the Ra3 values were still significantly higher than the Ra0 ( *p* < 0.05) and only Ra3 of the CJ group was comparable to the baseline ( *p* > 0.05).

**Table 3:** Means and standard deviations of surface roughness of the 5Y-PSZ at baseline (Ra0), after grinding (Ra1), after polishing (Ra2), and after high-gloss polishing (Ra3) (μm)

Groups		Mean surface roughness (SD; μm)				
Zirconia brands	Burs	Baseline (Ra0)	Grinding (Ra1)	Polishing (Ra2)	High-gloss polishing (Ra3)	*p* -value
Lava Esthetic	EVE (LE)	0.12(0.02) ^aA^	1.62(0.33) ^bA^	0.21(0.06) ^cAB^	0.18(0.03) ^cA^	< 0.001
	Jota (LJ)	0.12(0.03) ^aA^	0.74(0.12) ^bB^	0.13(0.06) ^aAB^	0.14(0.04) ^aAB^	< 0.001
Cercon xt	EVE (CE)	0.12(0.02) ^aA^	0.38(0.16) ^bC^	0.21(0.04) ^cAC^	0.18(0.03) ^cA^	0.001
	Jota (CJ)	0.12(0.04) ^aA^	0.38(0.07) ^bC^	0.18(0.06) ^cAB^	0.13(0.03) ^acB^	< 0.001
*p* -value		0.966	< 0.001	0.023	0.004	

Note: Different superscript lowercase letters indicate statistically significant difference among four different steps in the same row, analyzed by one-way repeated analysis of variance (ANOVA) followed by Bonferroni post hoc analysis ( *p* < 0.05).

Different superscript uppercase letters indicate statistically significant difference between groups in the same column, analyzed by one-way analysis of variance (ANOVA), followed by Bonferroni post hoc analysis ( *p* < 0.05).

When comparing different polishing protocols within the same material, the LJ group had significantly lower roughness values than the LE group in Ra1, Ra2, and Ra3 ( *p* < 0.05). On the other hand, there were no significant differences after the grinding and polishing steps between the CE and CJ groups ( *p* > 0.05) in Ra1 and Ra2. However, Ra3 of the CJ group was significantly lower than that of the CE group ( *p* < 0.05).

### Biaxial Flexural Strength

The mean and standard deviation of the biaxial flexural strength values are presented in Table 4 . In both zirconia products, the control groups had the highest flexural strength, that is, LC and CC. After grinding and polishing, the LE group was the only group with a significantly reduced flexural strength compared with the control groups ( *p* < 0.05). There was no difference in the flexural strength among the Cercon xt groups ( *p* > 0.05).

**Table 4:** Means and standard deviations of the biaxial flexural strength of the 5Y-PSZ (MPa)

Groups	Mean ± SD ( *n* = 60) Biaxial flexural strength (MPa)
**LC**	508.16 ± 34.01 ^a^
**LE**	423.77 ± 77.96 ^b^
**LJ**	486.69 ± 70.58 ^a,b^
**CC**	532.37 ± 15.73 ^a^
**CE**	513.82 ± 21.14 ^a^
**CJ**	516.40 ± 37.18 ^a^

Note: Different superscript lowercase letters indicate statistically significant difference among three different groups in the same material, analyzed by one-way analysis of variance (ANOVA) followed by Bonferroni post hoc analysis ( *p* < 0.05).

### Scanning Electron Microscope Analysis

**Fig. 3:**
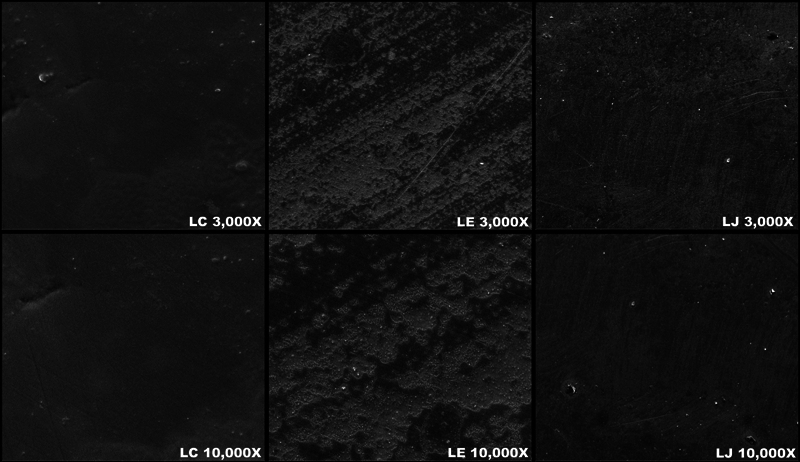
The scanning electron microscopy (SEM) images showed the surface topography of Lava Esthetic specimens (LC, LE, LJ) at 3,000X and 10,000X magnifications.

**Fig. 4:**
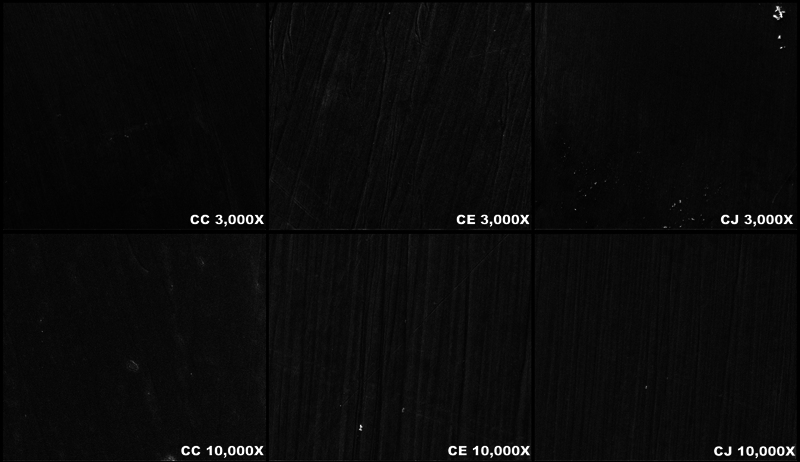
The scanning electron microscopy (SEM) images showed the surface topography of Cercon xt specimens (CC, CE, CJ) at 3,000X and 10,000X magnifications.

The microscopic surface topographies of all the tested groups at 3,000X and 10,000X magnification are shown in Figs. 3 and 4 . The LC and CC groups represent the control groups and appeared to have a smooth surface. The LE and CE groups showed rougher surfaces and scratches compared with the LJ and CJ groups.

### X-Ray Diffraction Analysis

**Fig. 5:**
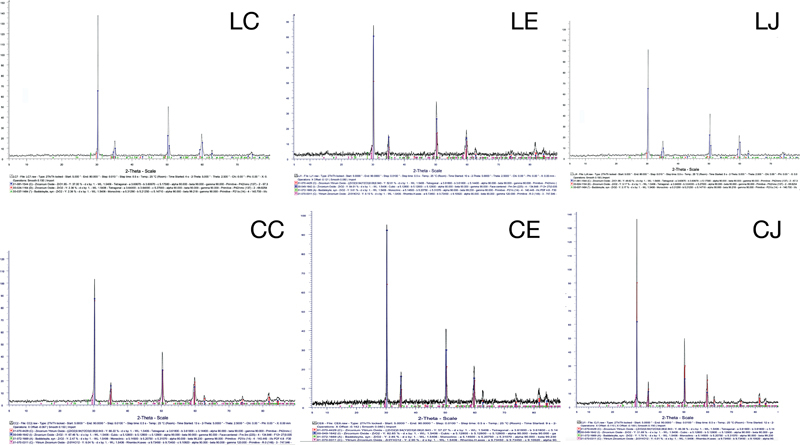
Representative X-ray diffractograms of Lava Esthetic specimens with the LC, LE, and LJ groups (left column) and Cercon xt specimens in CC, CE, and CJ group (right column). The diffractograms showed the view of peak from the 30 to 35°C and 50 to 62°C in all groups.

The XRD analysis is presented in Table 5 , with the corresponding XRD diffractogram shown in Fig. 5 . The LC and CC control groups exhibited comparable amounts of zirconia phases, with the tetragonal phase dominating at 69.82 and 64.92%, respectively, followed by the cubic and monoclinic phases. Following the grinding and polishing procedures, the LE and LJ groups showed an increase in the tetragonal phase compared with the LC group, while the cubic phase markedly decreased. This trend was also observed in the CC, CE, and CJ groups, with the tetragonal phase predominant, followed by the cubic and monoclinic phases. The percentage of the tetragonal phase in the CE and CJ groups increased to 91.32 and 93.31%, respectively, while the cubic phase decreased to 5.91 and 7.60%, respectively. However, the monoclinic phase remained comparably consistent across all groups.

**Table 5:** Composition of zirconia phase from XRD analysis

Groups	Tetragonal phase ( *t-* ZrO _2_ )	Monoclinic phase ( *m-* ZrO _2_ )	Cubic phase ( *c-* ZrO _2_ )
LC	69.82	0.75	29.43
LE	92.74	1.13	6.13
LJ	89.79	1.00	9.21
CC	64.92	0.73	34.35
CE	91.32	1.08	7.60
CJ	93.31	0.78	5.91

## Discussion

This study aimed to evaluate the effect of two grinding and polishing protocols on the surface roughness and biaxial flexural strength of 5Y-PSZ. The results revealed that both protocols led to changes in surface roughness and biaxial flexural strength of 5Y-PSZ. Therefore, the null hypothesis was rejected. After the zirconia restoration is fabricated, the grinding and polishing procedures, that is, occlusal adjustment, are inevitable in clinical situations. These procedures may generate heat and induce stress on the surface of zirconia, potentially leading to a transformation toughening phenomenon and iatrogenic damage to the restoration. However, previous studies revealed that the biaxial flexural strength was unaffected. 24 25

Two commercial 5Y-PSZ were chosen because of the improvement of its translucency by altering the grain size of zirconia. In addition, the fraction of the tetragonal and cubic phases is also altered, as the amount of cubic phase increases, while the tetragonal phase decreases. Consequently, the phase transformation toughening is unlikely to occur in 5Y-PSZ. 4

Currently, various zirconia polishing kits are available in the market, categorized based on bur speeds (slow and high speed) and different diamond particle sizes. The protruded platform on the tested specimen was designed to simulate a high-contact point of the restoration requiring adjustment in clinical situations. To maintain the properties of 5Y-PSZ and minimize the deterioration of the material, the selection of grinding and polishing burs for the adjustment of zirconia must be considered. The sequential polishing step should be applied to acquire an effective polished surface. 26 Diamond abrasive is commonly used as a primary component of zirconia polishing bur to improve the grinding efficiency due to the highest Mohs hardness scale. Therefore, two types of zirconia adjustment kits, slow-speed diamond burs (protocol I) and a high-speed superfine diamond bur followed by slow-speed diamond burs, were selected for evaluation (protocol II).

Many studies have investigated the effect of polishing procedures on zirconia with different polishing kits. However, only a few studies controlled the grinding and polishing protocols with custom polishing machines. 9 15 27 To minimize operator fatigue and errors during the procedures, in this study, the grinding and polishing protocols followed the manufacturer's instructions. The process progressed from large to small grain size of the burs in each step, utilizing a custom polishing machine to control direction, speed, number of polishing strokes, and coolant. The application force for grinding burs was regulated to 0.98 N to reflect the typical grinding force used by general dentists. 20 21 Moreover, the polishing burs were set to 2 N, which is considered the appropriate force for zirconia polishing. 22 23

The surface roughness of the restoration plays an important role in bacterial adhesion and restoration durability. 28 In this study, Ra0 was controlled as the baseline surface roughness value, comparable to the surface roughness value of the glazed surface of the ceramic restoration, 29 simulating the glazed surface of the restoration before starting the clinical adjustment procedure. The mean surface roughness of the materials was significantly increased with the rough grinding burs and started to decrease with the polishing and high-gloss polishing burs. This was corresponded with the SEM images that exhibited smoother and uniform surfaces after the high-gloss polishing. The roughness values after the final high-gloss polishing were within the clinically acceptable range of less than 0.20 μm, which could be susceptible to plaque accumulation. 30

Despite being the same 5Y-PSZ zirconia, containing 5%mol yttrium oxide, there was a significant difference after grinding, which could be attributed to the product compositions. Cercon xt contained hafnium oxide, aluminum oxide, and silicon oxide. In contrast, Lava Esthetic did not contain hafnium oxide. The incorporation of metal oxides in the composition provided the potential for enhancing physical and mechanical properties. An increase in hafnium concentration correlated with a reduction in porosity between the gain boundaries within the zirconia material. 31 32 Moreover, the resistibility of the material to withstand the abrasive particles of the grinding burs also affected the roughness values. It was suggested that the surface roughness highly depended on the microstructure of the ceramics, such as the types, configurations, and grain size. 33

Variations in the type, shape, size, density, and binding material of the diamond particle in the grinding burs also demonstrated different roughness values. 27 When comparing the two grinding and polishing protocols, the surface roughness after polishing with protocol I was significantly higher than that with protocol II. This could be explained by the slow speed and the large diamond particle size of the grinding bur that created a rougher surface of the material. However, this was not observed in Cercon xt, which could be due to the compositions of the material. Thus, it could be suggested that protocol II might be preferred for Cercon xt.

Despite the superior smoothness of the glazed surface, the longevity of the glaze was not well established after use in clinical situations. 12 Chairside adjustments normally removed the glaze layer from the restoration, leaving a rougher surface. The grinding procedure also resulted in an increased surface roughness value, as indicated by the increase in Ra1. Therefore, it is recommended to perform the polishing sequence step-by-step, starting with medium to fine diamond polishing burs after grinding to achieve the smoothest surface. 33

The mean flexural strength values obtained in this study were 423 to 532 MPa, which were nearly 460 to 630 MPa according to the value of the manufacturer's information and previous studies. 4 34 The flexural strength of both 5Y-PSZ in the control group exhibited the highest value. On the contrary, the groups that had undergone grinding and polishing procedures, that is, the LE, LJ, CE, and CJ groups, revealed decreased mean flexural strength values, which correlated with Iseri et al's study. 19 That study utilized low- and high-speed coarse grinding burs for grinding the 5Y-PSZ and observed an increase in the surface temperature of 5Y-PSZ. The results revealed decreased flexural strength values due to higher stress compression occurring on the high-temperature surface with both speeds of grinding burs. Moreover, a previous study also reported the effect of chairside adjustment using a coarse-grit 150-μm diamond bur without coolant. The flexural strength values significantly decreased compared with the unpolished 5Y-PSZ. 35 To explain this phenomenon, the authors speculated that the decrease in material strength was caused by surface defects such as microcracks and flaws on the tension surface. These defects were created during the grinding procedure and may not be completely removed even after high-gloss polishing. These factors could increase the origin of failure in the biaxial flexural strength, as observed in the results of this study.

The stabilized zirconia cannot undergo the t-m phase transformation due to the presence of the cubic phase, which prevents it from being affected by stress induced by environmental factors such as grinding, polishing, and sandblasting. 36 37 In clinical settings, occlusal adjustment could generate heat and force, potentially inducing phase transformation. 38 This change in the crystallographic microstructure of zirconia compromises the mechanical properties and longevity of the restoration. 39 The XRD result showed a reduction in the cubic phase and an increase in the tetragonal phase, indicating a reversal transformation. 40 This transformation was attributed to the internal structure of zirconia, affected by the martensitic transformation due to thermomechanical effects. However, there was no evidence supporting this phenomenon affecting the properties of the material. 41 The percentage of the monoclinic phase in the XRD showed a slight increase compared with the control, which was not related to the value of the biaxial flexural strength, similar to the results of Hatanaka et al's study. 42

Moreover, a previous study revealed that the grinding and polishing procedures may have also induced the rhombohedral phase fraction, which was a new phase formed under stress application from the cubic phase ( *c* -ZrO _2_ ) and tetragonal phase ( *t* -ZrO _2_ ) in 5Y-PSZ. 43 However, there was no consensus on whether this phase represents the true rhombohedral phase or a distorted form of the tetragonal and/or cubic phases. 43 44 From the XRD results, the rhombohedral phase was not detected, as only a minimal amount of this phase was present in the testing specimens. Furthermore, it is interesting to observe the translucency of 5Y-PSZ after grinding and polishing, which occurs due to the change from the cubic phase to the tetragonal phase.

The limitation of the study was the use of only two products of monolithic 5Y-PSZ. Nowadays, there are multiple brands of zirconia, including the multilayer type, and more grinding and polishing systems are available in the market, which need to be evaluated. Moreover, the limited number of specimens in the biaxial flexural strength test was insufficient for the required Weibull statistical analysis according to the ISO standard. 45 Further studies with larger sample sizes are needed for accurate test determination. Finally, an in-depth study of the zirconia structure needs to be conducted to provide detailed information on the material surface.

## Conclusion

As a result of this study, it is advised that the grinding and polishing protocol adhere to the manufacturer's instructions step by step, culminating in the final high-gloss polishing bur, to achieve the lowest surface roughness. Additionally, it was observed that the flexural strength of Lava Esthetic was affected by grinding and polishing following protocol I.
